# Optimized office lighting advances melatonin phase and peripheral heat loss prior bedtime

**DOI:** 10.1038/s41598-022-07522-8

**Published:** 2022-03-11

**Authors:** Marta Benedetti, Lenka Maierová, Christian Cajochen, Jean-Louis Scartezzini, Mirjam Münch

**Affiliations:** 1 Solar Energy and Building Physics Laboratory (LESO-PB), Ecole Polytechnique Fédérale de Lausanne (EPFL), 1015 Lausanne, Switzerland; 2 University Centre for Energy Efficient Buildings (UCEEB), Czech Technical University in Prague, Trinecka 1024, 27343 Bustehrad, Czech Republic; 3 Centre for Chronobiology, Psychiatric Hospital of the University of Basel, Wilhelm Klein-Strasse 27, 4002 Basel, Switzerland; 4 Transfaculty Research Platform Molecular and Cognitive Neurosciences, University of Basel, Basel, Switzerland; 5 Research Centre for Hauora and Health, Massey University, Wellington, New Zealand

**Keywords:** Neuroscience, Physiology, Endocrinology

## Abstract

Improving indoor lighting conditions at the workplace has the potential to support proper circadian entrainment of hormonal rhythms, sleep, and well-being. We tested the effects of optimized dynamic daylight and electric lighting on circadian phase of melatonin, cortisol and skin temperatures in office workers. We equipped one office room with an automated controller for blinds and electric lighting, optimized for dynamic lighting (= *Test room*), and a second room without any automated control (= *Reference room*). Young healthy participants (n = 34) spent five consecutive workdays in each room, where individual light exposure data, skin temperatures and saliva samples for melatonin and cortisol assessments were collected. Vertical illuminance in the *Test room* was 1177 ± 562 photopic lux (mean  ± SD) , which was 320 lux higher than in the *Reference room* (*p* < 0.01). Melanopic equivalent daylight (D65) illuminance was 931 ± 484 melanopic lux in the *Test room* and 730 ± 390 melanopic lux in the *Reference room* (*p* < 0.01). Individual light exposures resulted in a 50 min earlier time of half-maximum accumulated illuminance in the *Test* than the *Reference room* (*p* < 0.05). The melatonin secretion onset and peripheral heat loss in the evening occurred significantly earlier with respect to habitual sleeptime in the *Test* compared to the *Reference room* (*p* < 0.05). Our findings suggest that optimized dynamic workplace lighting has the potential to promote earlier melatonin onset and peripheral heat loss prior bedtime, which may be beneficial for persons with a delayed circadian timing system.

## Introduction

Daily exposure to light is essential for stable entrainment of the human circadian pacemaker to the natural 24-h light/dark cycle^1–4^. Office workers spend the majority of their work time within buildings^5–7^, where lighting quantity and quality might not be adequate enough to elicit a sufficiently strong signal for entrainment of internal clocks with the external 24-h day. Exposure to low lighting during daytime and too much electric lighting in the evening can both facilitate delays in the human circadian timing system, leading to a mismatch between endogenous circadian time (e.g. body time) and social time (e.g. work times). Such mismatch is negatively affecting body and brain functions, including sleep, physical and cognitive performance^8–11^. Despite recent recommendations for lighting^12–14^, it still remains to be elucidated, how much light is needed for optimal synchronization and stable entrainment of the circadian clock(s) with the 24-h day especially at the individual level and under daily life conditions.

Rather few studies were done in naturalistic, real-life settings^15–24^. Among those, Wright and colleagues conducted several studies with only daylight exposure and lighting from fireplaces while camping. They compared the results of melatonin secretion patterns and sleep timing of the same study participants when they were living and working in their habitual indoor environments, with less light during the day and additional electric light in the evening^22^. The authors found that exposure to natural light during the day led to a circadian phase-advance of the onset of melatonin secretion in the evening of about 2 h (compared to habitual mixed electric and daylighting conditions), which was more pronounced in later than earlier chronotypes. Living under natural light conditions also prolonged the phase angle of entrainment defined as the temporal relationship between the dim light onset of melatonin (circadian phase marker) in the evening and sleep onset by 49 min (see supplementary information of Ref^22^).

Non-visual light effects in humans are often quantified by measuring acute melatonin suppression and circadian phase shifts of the dim light melatonin onset (DLMO) in response to light^25,26^. However, the effects of prior light exposure during the day, which additionally modulate acute effects of light in the evening (e.g. cognitive performance^27–29^ melatonin suppression^15,30,31^, circadian phase shifts^30,32,33^, circadian amplitude^34^, alertness^29,35^ and sleep^36–38^) have much less been considered. Bright light exposure during the day increased nocturnal peak levels in melatonin^39,40^, and exposure to 5000 photopic lux (at eye level) at midday for three consecutive days significantly phase-advanced the onset of melatonin secretion^41^. On the other hand, if prior daytime light exposure was low, bright light in the evening led to stronger melatonin suppression and greater delays in melatonin phase^15,30,31,42^.

Regarding the impact of light exposure on the circadian modulation of cortisol concentrations, bright light in the early morning increased the morning peak^43,44^, advanced the circadian phase of cortisol^34,45^ and acutely suppressed cortisol concentrations (after 6.7 h bright light exposure, starting around the melatonin peak at night)^46^.

Core body temperature (CBT) regulation is also modulated by the endogenous circadian clock^47,48^. The circadian rhythm of CBT exhibits a maximum in the late afternoon and a minimum 2–3 h before habitual waketime^3,47,49^. Bright light exposure does not only shift the rhythm of melatonin secretion but also CBT in a phase-dependent manner^50^ and depends on spectral power distribution of light^51^. There is a strong interconnection between melatonin secretion in the evening and peripheral heat loss via hands and feet which precedes the CBT decline in preparation for nocturnal sleep^52^. Such thermoregulatory modulation can be determined by the distal–proximal skin temperature gradient (DPG)^53^, and DPG can thus be considered as another indirect circadian phase marker.

Our aim was to investigate the effects of optimized dynamic office lighting with mixed day- and electric light on the circadian phase of salivary melatonin onset and DPG in the evening as well as cortisol concentrations in healthy young participants. Compared to a reference condition, we expected that the optimized dynamic lighting system enables high daytime illuminance (at all weather conditions) with phase advancing effects (e.g. on melatonin secretion) under habitual home lighting conditions in the evenings. Thus, we performed a semi-naturalistic study where participants spent one week (i.e., five working days between 8:00 and 18:00) in an office room with an automated control for dynamic lighting (daylight and electric light), designed to optimize biological light effects, and one week in a reference room with mixed daylight and electric lighting but without a control system. This work uses some of the content of the PhD thesis of Dr. Benedetti^54^.

## Results

### Vertical illuminance measured by stationary light sensors in the *Test* and *Reference room*

**Figure 1:**
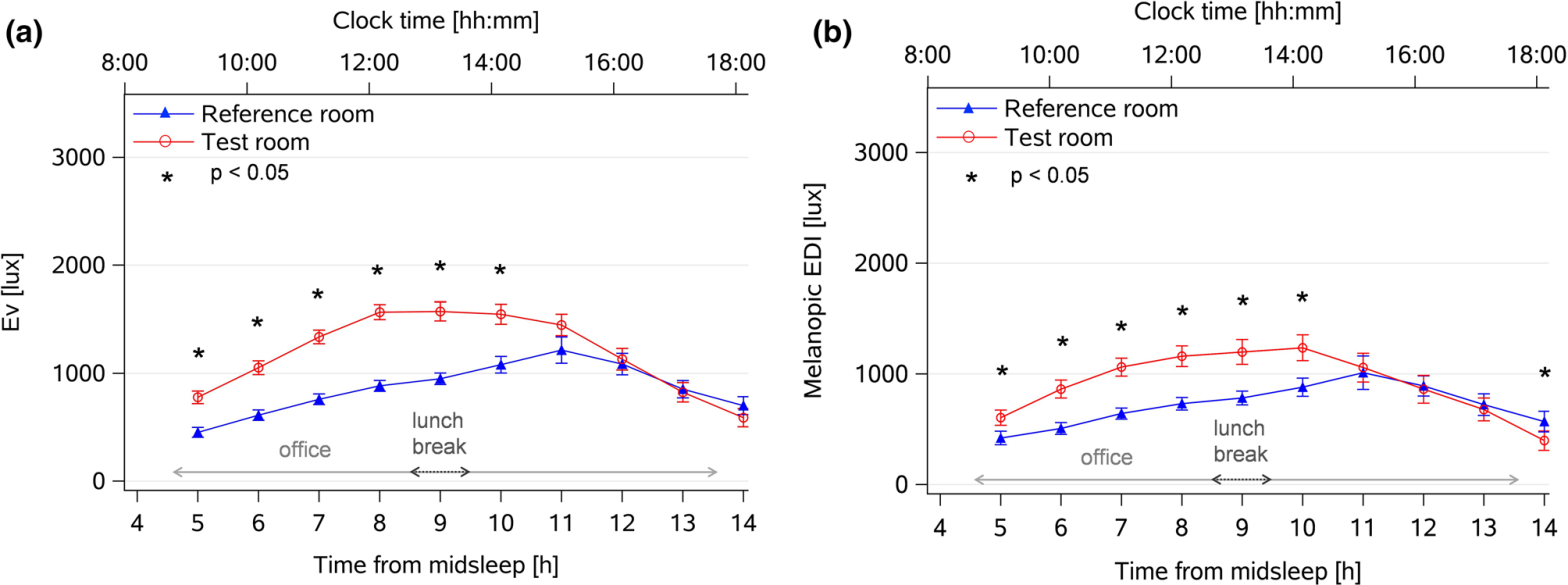
Time course of vertical illuminance (E_v_) and melanopic EDI during office hours from stationary light sensors. (**a**) Time course of vertical illuminance (E_v_, lux) measured by stationary light sensors at the desk [at 1.2 m height from floor on a vertical plane at the approximate eye level of a sitting person (averaged across 5 days during office hours; n = 31 for the *Reference* and n = 34 for the *Test* room)]. (**b**) Averaged melanopic Equivalent Daylight (EDI) Illuminance (lux) at the desk (mean values ± SEM, n = 18; see text for more information). Symbols: *Test room* = red lines and circles; *Reference room* = blue lines and triangles (mean ± standard error; SEM)]; all values are aligned to elapsed time since habitual midsleep (lower x-axis). The upper x-axis also shows averaged clock times. The office sessions (indicated by the light grey vertical arrows) started approximately one hour after habitual waketime, and ended in the later afternoon. The dotted grey horizontal arrows indicate the lunch break (spent outside the office); * = significant differences between both conditions (with *p* values adjusted for multiple comparisons).

Vertical illuminance measured by a stationary light sensor (E_v_; lux; measured next to a sitting participant in a vertical plane at eye level), during scheduled office hours varied significantly over time in both rooms (n = 34; Fig. 1a, main effect of *time*: F_12,564_ = 36.2, *p* < 0.0001) and was on average significantly higher in the *Test* than the *Reference room*: 1177 ± 562 photopic lux versus 858 ± 478 photopic lux, (mean ± SD; Table 1; main effect of *condition:* F_1,599_ = 54.9, *p* < 0.0001). An interaction with the factors condition*time (F_11,563_ = 8.7, *p* < 0.0001) and post-hoc comparisons (with *p* values adjusted for multiple comparisons, see Statistics) revealed significantly higher E_v_ levels in the *Test room* between 5 and 10 h since habitual midsleep time (derived from 7 days before each week spent in the office, see “Methods”), which corresponded to average clock times between 9:00–14:00 approximately (*p* < 0.047).

**Table 1:** Summary of lighting characteristics assessed during the study derived from stationary and wearable light sensors.

Condition	Photopic E_v_ [lux]	Melanopic EDI [lux]	Individual E_v_ (wearable light sensor) [lux]	
	Office hours	Office hours	Office hours	All day (from waketime to bedtime)
Reference room	858 ± 478 (n = 34)	730 ± 390 (n = 18)	1036 ± 1518 (n = 33)	675 ± 1351 (n = 33)
Test room	**1177 ± 562** (n = 31)	**931 ± 484** (n = 18)	1018 ± 1246 (n = 30)	650 ± 1132 (n = 30)

Light exposure measurements: Stationary light sensors: E_v_ = photopic illuminance, EDI = melanopic equivalent (D65) daylight illuminance (lux), both assessed on a vertical plane at the approximate eye level during office hours. Wearable light sensors: individual E_v_ during office hours and during entire waking period (mean ± SD per hour across days and participants). Bold numbers (for photopic E_v_ and melaonpic EDI) indicate significantly higher values in the *Test room* than the *Reference room* (*p* < 0.05).

### Melanopic Equivalent Daylight Illuminance measured by stationary light sensors in the *Test* and *Reference room*

Melanopic Equivalent Daylight Illuminance (melanopic EDI, lux; measured by a stationary device next to a sitting participant in a vertical plane at eye level), varied significantly during scheduled office hours in both room conditions (n = 18, see “Methods”; Fig. 1b, main effect of *time*: F_9,291_ = 19.6, *p* < 0.0001). However, there was a higher melanopic EDI in the *Test* than in the *Reference room* (931 ± 484 vs. 730 ± 390 melanopic lux; Table 1; main effect of *condition*: F_1,311_ = 16.6, *p* < 0.0001) and a significant interaction with the factors condition*time (F_9,291_ = 5.2, *p* < 0.0001). Post-hoc tests revealed significantly higher melanopic EDI in the *Test room* in the morning between 5–10 h after habitual midsleep and at the end of the office day (14 h after habitual midsleep) which corresponded to clock times between 9:00–14:00 and at 18:00 (*p* < 0.015).

### Individual light exposure measured by wearable devices at the participants’ eye level during the entire waking period

**Figure 2:**
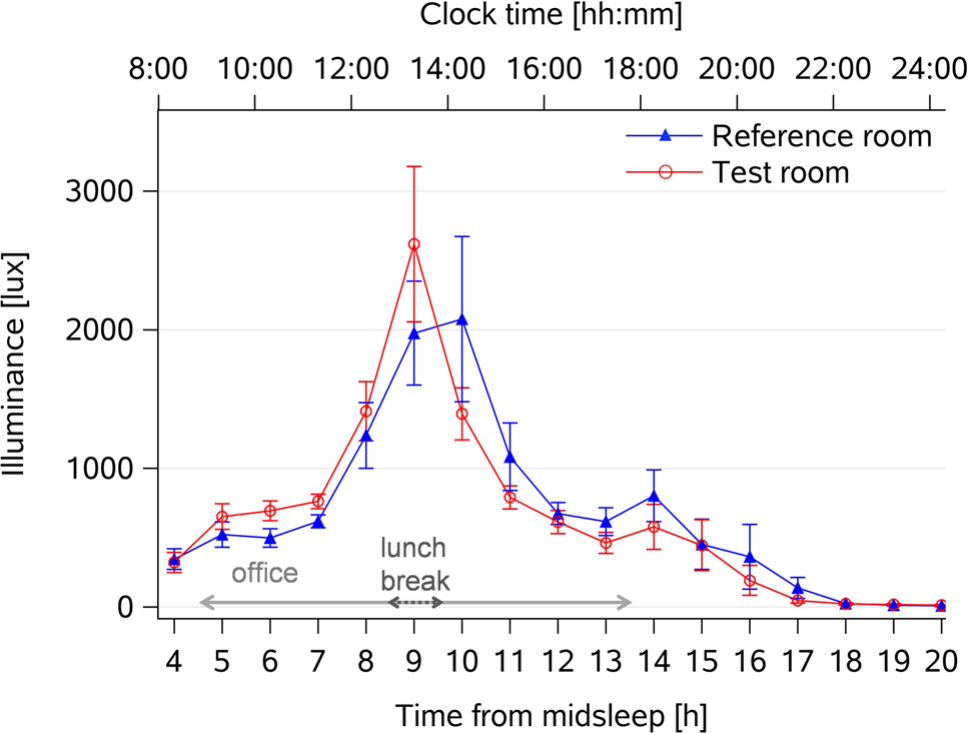
Individual light exposure (E_v_) from wearable light sensor devices across the entire waking period. Time course of averaged individual illuminance (E_v_) from wearable light sensors recorded across the entire waking period, averaged across 5 days (mean values ± SEM; *Test room* = red lines and circles, n = 30; *Reference room* = blue lines and triangles, n = 33). The x-axis at the bottom shows elapsed time since habitual midsleep (h), and the upper x-axis depicts corresponding clock times. The horizontal arrows show the time in the offices (light grey arrow) and the lunch break (spent outside the office; dotted grey arrow).

The time course of individual photopic illuminance, measured by wearable devices at the eye (E_v_) was continuously measured between habitual wake- and bedtimes. It varied over time for both room conditions (main effect of *time:* F_16,998_ = 173.4, *p* < 0.0001; Fig. 2), but in contrast to the E_v_ measured next to the participant during office hours (see 2.1), it revealed no statistically significant differences (or interactions with the factors condition*time) between the two room conditions (averaged across days between wake- and bedtimes; *p* > 0.5). It was 650 ± 1132 lux (mean ± SD) for the *Test room* and 675 ± 1351 lux for the *Reference room* (Table 1).

From visual inspection however, it appeared that there may be illuminance differences between both room conditions only in the first half of the day. Thus, we analyzed individual E_v_ from 5 to 9 h after midsleep (i.e., between the time of arrival in the office to lunch time). The results indeed showed significantly higher individual photopic E_v_ in the *Test* (1231 ± 1665 lux) than in the *Reference room* (973 ± 1300 lux; main effect of *condition*: F_1,289_ = 10.1, *p* = 0.002), with a variation over time (main effect of *time*: F_4,267_ = 38.2, *p* < 0.0001), but no significant interaction between both factors (*p* > 0.5). When the same analysis was carried-out with the photopic E_v_ for the second half of the day (i.e., after 10 h since midsleep until bedtime), there was no main effect of *condition* (*p* > 0.1).

**Figure 3:**
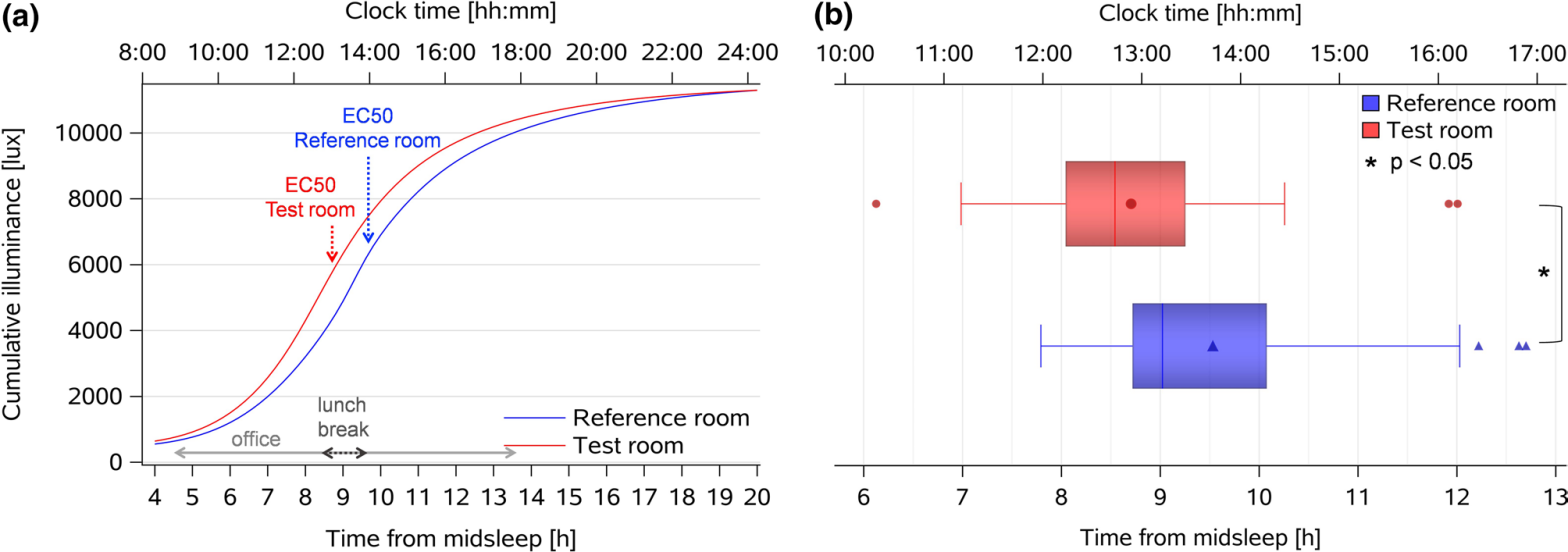
Dose–response curves for accumulated individual illuminance (E_v_) from wearable light sensors and half-maximum responses (EC50). (**a**) Dose–response curves for accumulated individual vertical illuminance (E_v_) from wearable light sensors, aligned to elapsed time since habitual midsleep (lower x-axis) and corresponding clock time (upper x-axis); vertical arrows indicate the half-maximum response times [EC50; mean = 8:42 (h:mm) since habitual midsleep in the *Test room* (red; n = 30), and 9:32 since habitual midsleep in the *Reference room* (blue, n = 33)]; the horizontal arrows show the time in the offices and the lunch break spent outside the office. (**b**) Box plots for half-maximum response times of individually accumulated illuminance (E_v_). The box plots show mean (red and blue symbols inside the boxes), median (red and blue vertical lines), minimum and maximum values (red and blue horizontal bars), outliers (symbols outside the boxes).

In the next step we computed the accumulated photopic E_v_ in both conditions by applying non-linear curve fitting on individual curves to evaluate whether the timing of accumulated E_v_ was different between both conditions (see “Methods”). The results from the individually fitted curves confirmed that the half-maximum response (EC50) of accumulated E_v_ occurred significantly earlier in the *Test room* than in the *Reference room* i.e., at 8:42 ± 1:16 versus 9:32 ± 1:22 (h:mm) since midsleep (main effect of *condition:* F_1,33_ = 5.4, *p* = 0.027; Fig. 3a,b) corresponding to clock times 12:57 and 13:35 (please note that clock time differences slightly deviate due to the non-significant average clock time differences of 12 min between the *Test* and the *Reference* *room* condition, see Table 2(a) and “Methods”). The accumulated E_v_ at EC50 was on average 5950 photopic lux in the *Test room*, which corresponds to ~ 4800 lux melanopic EDI.

**Table 2:** (a) Summary of habitual sleep times and (b) melatonin secretion onset and offset times, DPG rise/decline and phase angles.

Condition	Reference room	Test room	*p* value
	**Clock time [hh:mm]** **Mean ± SD**		
**(a)**			
Midsleep	04:02 ± 00:39	04:14 ± 00:40	0.23
Waketime	07:58 ± 00:36	08:05 ± 00:48	0.86
Sleep onset	00:05 ± 00:56	00:23 ± 00:46	0.66
Sleep duration	07:53 ± 00:52	07:41 ± 00:51	0.44
	**Time since habitual midsleep [hh:mm, mean ± SD]** **(Clock time; [hh:mm, mean ± SD]) #**		
**(b)**			
Time of half-maximum dose of accumulated E_v_	09:32 ± 01:22 (13:35 ± 01:01)	08:42 ± 01:16 (12:57 ± 01:03)	**0.027**
Melatonin secretion onset	17:05 ± 1:29 (21:07 ± 01:31)	16:40 ± 01:18 (20:54 ± 01:18)	**0.048**
Melatonin secretion offset	05:02 ± 00:56 (09:04 ± 01:01)	04:56 ± 00:54 (09:11 ± 01:00)	0.62
DPG rise	18:56 ± 01:08 (22:58 ± 1:30)	18:36 ± 00:56 (22:48 ± 01:07)	0.10
DPG decline	03:53 ± 00:44 (07:55 ± 00:50)	03:34 ± 00:33 (07:47 ± 00:46)	**0.011**
	**Time [hh:mm] Mean ± SD**		
Phase angle melatonin onset = (sleep onset minus melatonin secretion onset)	02:58 ± 01:41	3:29 ± 01:22	**0.009**
Phase angle DPG rise = (sleep onset minus DPG rise)	01:07 ± 01:00	01:34 ± 01:00	**0.042**
Phase angle DPG decline = (waketime minus DPG decline)	00:02 ± 00:28	00:15 ± 00:26	**0.049**

(a) Habitual midsleep time, waketime, sleep onset time and sleep duration (hh:mm), mean per condition ± SD (derived from activity watches) are shown for both conditions (derived from the 7 nights prior the laboratory week and were used for calculation of melatonin and DPG onset, offset times and phase angles during both room conditions in (b)); (b) time of half-maximum dose of accumulated E_v_, melatonin onset and offset times, DPG rise and decline times since midsleep (these times are also indicated in clock times, in brackets). Phase angle durations are shown for time ranges between: melatonin onset time and sleep onset time; DPG rise time and sleep onset time; DPG decline time and waketime. Significant *p* values are shown in bold; # clock times derived from habitual midsleep time for each condition separately; average midsleep time differed by 12 min between the two conditions (*p* = 0.23).

### Melatonin and cortisol

#### Melatonin

Salivary melatonin secretion profiles during habitual waketime were averaged across two days (see also below) and per room condition and exhibited a typical time course (supplementary Figure S5a; main effect of *time*: F_10,565_ = 123.2, *p* < 0.0001), with no significant concentration differences between both conditions (*p* > 0.1).

**Figure 4:**
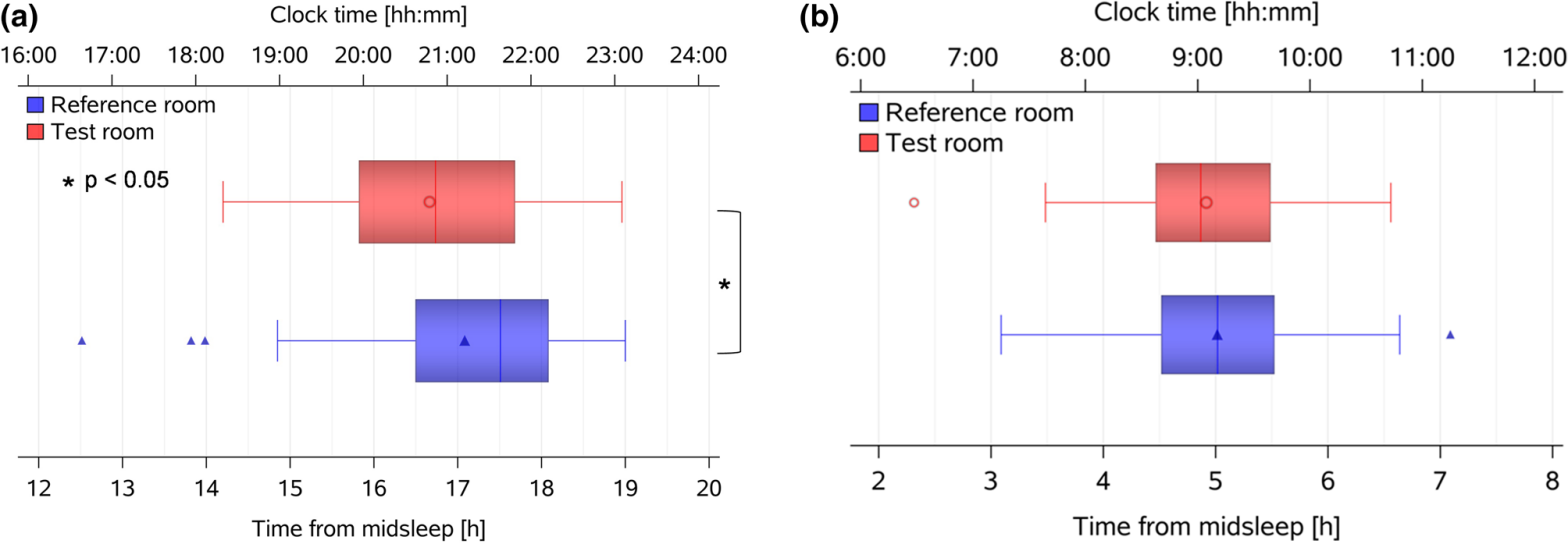
Melatonin secretion onset and offset times. Box plots for (**a**) melatonin secretion onset and (**b**) melatonin secretion offset, averaged across 2 days per condition, and aligned to elapsed time since habitual midsleep for both conditions (corresponding clock time is shown on the upper x-axis). Melatonin onset occurred significantly earlier in the *Test room* (red symbols; n = 31) than in the *Reference room* (blue symbols; n = 34; *: *p* = 0.048). There was no significant difference between both conditions for melatonin offset time. The box plots show mean (red and blue symbols inside the boxes), median (red and blue vertical lines), minimum and maximum values (red and blue horizontal bars).

Melatonin onsets at home (defined as time when salivary melatonin concentrations exceeded a threshold of 3 pg/ml, see “Methods”) were not statistically different between the first and the second day of salivary collections (*p* > 0.5) and therefore, all melatonin analyses were performed on averaged melatonin secretion profiles per condition. Melatonin onset occurred significantly earlier during the week in the *Test* than the *Reference room* (Fig. 4a; main effect of *condition*: F_1,31_ = 4.2, *p* = 0.048), i.e., 16:40 ± 1:18 (hh:mm) versus 17:05 ± 1:29 since habitual midsleep, which corresponds to clock times at 20:54 ± 1:18 and 21:07 ± 01:31 (see Table 2(b)).

For the melatonin offset (averaged for both sampling days, see “Methods”), there were no significant differences between both conditions (i.e., 4:56 ± 00:54 since habitual midsleep in the *Test room* versus 5:02 ± 00:56 since midsleep in the *Reference room*, corresponding to clock times 9:11 ± 01:00 and 9:04 ± 1:01, respectively; Fig. 4b). As a consequence of the earlier melatonin secretion onset and because participants were instructed to keep bedtimes constant during the study, phase angles of entrainment between melatonin secretion onset and habitual sleep onset (derived from rest-activity recordings preceding the laboratory week) became 31 min longer in the *Test room* than in the *Reference room* (*p* = 0.009), while habitual sleep timing was not significantly different (*p* > 0.5; see Table 2(b)).

#### Cortisol

As expected, we observed significant variations of cortisol concentrations during waking which were highest after habitual wake time (averaged across both sampling days; main effect of *time*: F_10,593_ = 85.6, *p* < 0.0001; supplementary Figure S5b), with no statistically significant differences between conditions, or interactions between the factors condition*time, even when we analyzed the first 4 data points after waketime separately (= morning values; *p* > 0.5).

### Distal–proximal skin temperature gradient

The 24-h time course of DPG profiles were averaged across 5 days (see supplementary Figure S6), it showed a significant diurnal variation (main effect of *time*: F_23,1454_ = 36, *p* < 0.0001). The DPG was overall significantly larger for the week in the *Test* than in the *Reference room* (2.35 ± 1.9 °C vs. 2.25 ± 1.8 °C, main effect of *condition*: F_1,1466_ = 4.1, *p* = 0.043), but there was no statistically significant interaction between the factors condition*time (*p* > 0.5).

**Figure 5:**
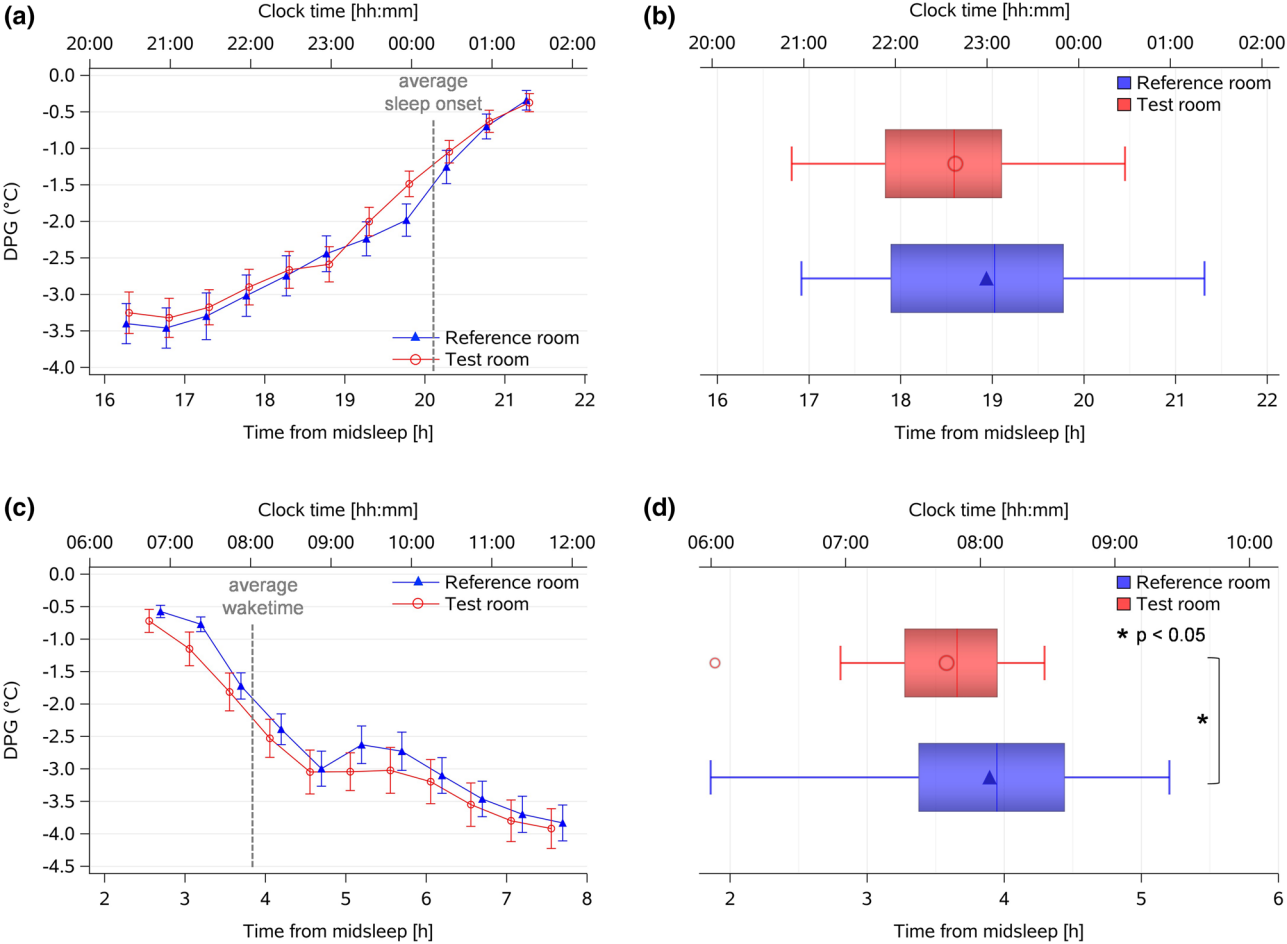
Evening rise and morning decline of DPG. (**a**) Averaged time-course of evening DPG, from 4 h before habitual sleep onset to 1 h after habitual sleep onset (mean ± SEM; red symbols = *Test room*, n = 30; blue symbols = *Reference room*; n = 34); the vertical dashed lines show averaged sleep onset for both conditions; (**b**) Box plots of the DPG rise in the *Test room* (red symbols) and in the *Reference room* (blue symbols; *p* = 0.10). The box plots show mean (red and blue symbols inside the boxes), median (red and blue vertical lines), minimum and maximum values (red and blue horizontal bars). All times are aligned to habitual midsleep time, and corresponding clock time is shown on the upper x-axis; (**c**) Averaged time-course of the DPG decline in the morning, from 1 h before habitual waketime to 4 h after habitual waketime for both conditions separately (red circles and lines = *Test room*, n = 30, blue triangles and lines = *Reference room*, n = 34; mean ± SEM); the vertical dashed line shows averaged habitual waketime for both conditions; (**d**) Box plots of DPG decline in *Test room* (red symbols) and in *Reference room* (blue symbols, *: *p* = 0.01).

**Figure 6:**
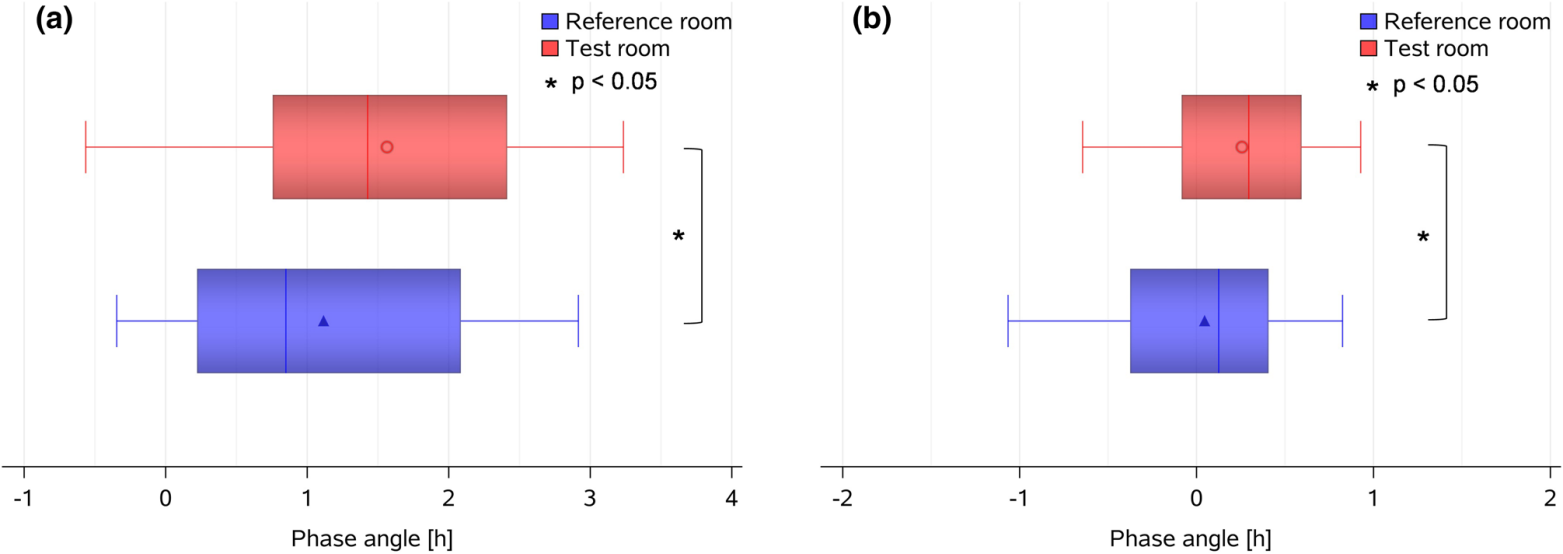
Phase angle between DPG rise time and sleep onset and DPG decline time and waketime. (**a**) Phase angle between DPG rise time and sleep onset (= sleep onset minus DPG rise time) in *Test room* (red symbols, n = 30) and in *Reference room* (blue symbols, n = 34; *: *p* = 0.042); (**b**) Phase angle between DPG decline time and habitual waketime (calculated as habitual waketime minus DPG decline) in *Test room* (red symbols, n = 30) and in *Reference room* (blue symbols, n = 34; *: *p* = 0.049); the box plots show means (red and blue symbol inside the box), median (vertical red and blue lines), minimum and maximum values (horizontal red and blue bars).

In a next step, we determined the exact rise times of the individual DPGs in the evening and DPG decline in the morning hours relative to habitual midsleep (for details see “Methods”). The evening rise of the DPG tended to occur earlier in the *Test* than in the *Reference room* (difference = 20 min, Table 2(b); Fig. 5a,b; *p* = 0.10). The phase angle between the time of the DPG rise and habitual sleep onset (derived from activity watches during seven days prior each laboratory part) became significantly longer in the *Test* than in the *Reference room* (difference = 27 min; main effect of *condition*: F_1,34_ = 4.5, *p* = 0.042; Fig. 6a, Table 2(b)). Similarly, the DPG decline in the morning occurred 19 min earlier in the *Test room* than in the *Reference room* (main effect of *condition*: F_1,32_ = 7.2, *p* = 0.011; Fig. 5c,d), whereas the phase angle between the time of the DPG decline in the morning and waketime became 13 min longer in the *Test room* than in the *Reference room* (*p* = 0.049; Fig. 6b).

### Sleep times during the weeks in the office

When we compared the real achieved midsleep, sleep onset and wake times as well as sleep durations between the *Test* and the *Reference room* during the week in the office, there were no significant differences between both room conditions (*p* > 0.2). But when these times were compared to those during the week prior each room condition, they occurred slightly but significantly earlier and there was also a shorter sleep duration (*p* < 0.001; see supplemental Table S4). The differences between sleep times and sleep duration prior and during the week in the office were not significantly different between both conditions either (*p* > 0.1), indicating that there was a similar advance of sleep times for the *Test* and the *Reference room* during the weeks in the laboratory.

## Discussion

We explored the effects of an automated dynamic lighting control on three circadian markers, melatonin, cortisol, and DPG, by optimizing mixed daylight and electric lighting during office hours. The photopic E_v_ (assessed by wearable light sensors) was higher during the first half of the day, and half-maximum response times of accumulated E_v_ were significantly earlier in the *Test* than in the *Reference room.* Concomitantly, the timing of melatonin onset and peripheral heat loss (DPG) relative to habitual sleep times occurred significantly earlier in the *Test* than in the *Reference room.* These results suggest that the use of an automated controller together with an optimized lighting system during the day has the potential to advance circadian phase markers, even under semi-naturalistic conditions in the presence of electric light in the evening.

Despite high E_v_ in both rooms (exceeding 500 photopic lux during most of the working hours), E_v_ was significantly higher in the first half of the office day in the *Test* than the *Reference room*. In both rooms, the light pattern changed dynamically, also because of the vast daylight availability. Melanopic EDI was at least 350 melanopic lux, which is in accordance with recent recommendations, where a minimum of 250 melanopic lux (during daytime) is advised^12^. Melanopic EDI was higher in the *Test* than the *Reference room*, particularly during the first half of the day (from 10:00 to 14:00). Interestingly, individual light exposures, measured by wearable light sensors at eye level, did not really reflect such large differences (even though E_v_ measured with those sensors was also higher during the first part of the day in the *Test* than the *Reference room*), presumably due to some impact from the angle of gaze/head movements, confirming existing study findings^55–58^.

Relative to habitual midsleep, there was a significant advance of melatonin onset, but not offset, which is interesting since the DPG offset and the DPG phase angles were significantly advanced/prolonged in the *Test* when compared to the *Reference room*. One reason might be that the melatonin offset is more variable, which has been shown in previous literature under stringent and controlled laboratory conditions^59^. The same study also showed that room light exposure (200 photopic lux in the horizontal angle of gaze) 8 h before bedtime suppressed melatonin secretion and shortened melatonin secretion duration by 90 min when compared to dim light (< 5 lux)^59^. In our study, neither evening nor morning light at homes were significantly different between both weeks in different office room conditions (and were on average 365 photopic lux in the morning before participants came to the office, and 205 photopic lux in the evening, i.e., during the last 5 h before bedtime). However, different lighting conditions during the day may have desensitized/sensitized participants differently for similar (room) light conditions in the evening (see^15,29–31,33,35^), a factor which added complexity to a so far unknown extent.

Since we aimed at measuring the onset of melatonin secretion in the evening under habitual light conditions at home and not under constant dim light conditions, our results were masked to a certain extend by environmental lighting, which most likely delayed the endogenous melatonin secretion onset. Thus, the actual melatonin secretion onset under dim light conditions (DLMO) would have occurred earlier, as it was shown by Wehr et al.^60^, who compared the onsets under real life lighting conditions and in dim light in the laboratory. Retrospectively, there was no statistically significant difference of individual E_v_ between both conditions in the last 5 h before bedtime in our within subject design (mean E_v_ *Reference room* = 172 lux and *Test room *= 131 lux; *p* = 0.699). Thus, it seems rather unlikely that the observed differences in circadian phases (e.g., of melatonin onset) were mainly due to different evening lighting conditions, despite some larger intra-individual differences in a few participants (see supplemental Figure S8).

Even though participants were instructed to keep bedtimes constant within a target bedtime of ± 30 min throughout the study, the real achieved sleep times during both weeks in the laboratory were slightly but similarly advanced when compared to habitual sleep times in the week prior. Whether this advance was also a consequence of the office lighting conditions (which were bright in both rooms) and partly contributed to the significant advance of the circadian phase in the *Test room* cannot be excluded.

We also found a significant correlation between earlier melatonin onsets and earlier rises of DPGs only for the *Test room* condition (*p* = 0.026; r = 0.3; data not shown). The correlation went in the same direction within participants, which makes a circadian phase advance under real life conditions very likely, despite similar (and masking) room lighting conditions in the evening in both conditions. If the optimized lighting would be used constantly during work hours, we would indeed expect a stronger phase advance of the melatonin onset and most likely also a general advance in sleep timing. This shift would certainly differ on weekends, where often a phase delay occurs, particularly in young adults. Such optimized lighting control may therefore be useful to re-entrain the circadian clock in young adults after the weekend. Thus, our lighting solution ideally synchronizes the endogenous circadian timing system with the daytime work-schedules.

The observed circadian phase advance of melatonin onset and DPG rise under semi-naturalistic office conditions in healthy young participants shows promise also in clinical settings, to treat patients with delayed sleep phase syndrome (DSPS)^61^. Despite the fact that real achieved sleep times were advanced in both room conditions, we could not observe more advanced sleep times for the *Test* than the *Reference* condition, since we asked the participants to keep them constant throughout the study. Our results may support findings from the literature, where it was shown that brighter morning light exposures can advance circadian phase^62,63^ (including sleep/wake timing) and is an effective treatment for DSPS^64,65^. Thus, translation of our findings into practice by using intelligent ‘phase advancing’ office lighting, enabled by automated controllers, could be an asset at workplaces in the future.

Another application would be in schools or universities. There is increasing evidence that teenagers, adolescents and healthy young adults commonly use bright screens and other light sources in the evening, which lead to circadian phase delays^66^, late sleep timing^67–70^ and may reinforce existing, or even trigger manifestation of circadian rhythm disorders. Low light levels during the day additionally exacerbate light responses in the evening, i.e., intensify non-visual responses such as prolonged sleep onset^20^, even though these evening light levels are not necessarily very bright. It was shown that daylight or morning light with greater portions of short-wavelengths lighting can be used as a countermeasure to mitigate such negative phase delaying effects of light in the evening, since it reduces the magnitude of phase shifts^33,71–73^, probably by desensitization effects. Late chronotypes and maybe also patients suffering from DSPS could benefit from the presented optimized office lighting control, as it favors an earlier circadian phase and prevents further delays, even more effectively if combined with limited exposure to shorter wavelengths of light and/or bright light in the evening^74^.

In the morning, we also found a significant earlier decline of DPG, which has been shown to facilitate sleep pressure dissipation^75^. We also would expect an earlier cortisol peak time in the morning, which we did not observe, partly due to the lack of a 24-h sampling period and thus, in some of the participants the cortisol peak may occurred before the first salivary sample.

Limitations of our study are certainly linked to the semi-naturalistic approach: there were no constraints on light exposures out of the office, for instance in the evening hours, or during the lunch break outside the building; there were no instructions for physical activity or meal timing, which may have contributed to individual variability of hormonal and light exposure data between conditions and between participants. In the same vein, during the week in the *Reference room*, participants could use the blinds and electric lighting according to their individual needs. It is to note that despite these individual allowances, there was no statistically significant difference of evening light exposures (assessed from the wearable light sensors, see also above).

To conclude, here we show that a customized mixed electric-daylighting control for offices can provide dynamically changing lighting patterns, which are typical for daylight—i.e., with large light fluxes in the morning and lower ones towards the end of the working day. More importantly, our results give evidence that such office lighting, regulated by an automated control system, can advance the circadian phase of melatonin and DPG. Late chronotypes and maybe also patients suffering from DSPS could benefit from the presented optimized office lighting control, as it favors an earlier circadian phase and prevents further delays.

## Methods

### Experimental set-up

The study was performed in two office rooms at the Laboratory for Solar Energy and Building Physics (École Polytechnique Féderale de Lausanne, Switzerland) with mixed natural and electric lighting: one room (= *Test room*) was optimized with regards to light’s biological effects (see more details in the supplement) in comparison to a common office room (= *Reference room*), where no such optimization was implemented. A customized programmed control system for the blinds and electric lighting was applied only in the *Test room* (see supplement). In the *Reference room*, no automated control was used, the user was free to adjust the electric lighting and shading manually according to his/her preferences. In addition, the upper part of the anidolic daylighting system^76^ was covered in the *Reference room* to mimic an ordinary office room.

The desks of each room were located next to the windows (supplementary Figure S1). Windows were equipped with motorized venetian blinds; both rooms were supplied with dimmable LED light sources on the ceiling and a standing floor lamp (see lamp types and spectra of the electric lighting, based on recent guidelines^77^ in the supplementary Table S2 and Figure S2).

### Participants

Participants were recruited at local Universities (École Polytechnique Féderale de Lausanne and University of Lausanne, Switzerland) by means of flyers and online announcements. All participants completed 4 screening questionnaires as well as one entry questionnaire and underwent a thorough interview (see supplement for inclusion criteria and Table S1 for results from screening questionnaires).

A total of 34 healthy participants completed the study: 18 females and 16 males (age: 23.4 ± 3.2 years; mean ± SD; see supplementary Table S1). Due to technical issues with the controller, the data of the *Test room* had to be excluded for three participants (out of five), two of those participants repeated the *Test room* condition. Data from the wearable light sensor could not be used due to technical problems with the sensor for one participant in the *Test room* and for another participant in the *Reference room*.

Finally, data of 31 participants for the *Test room* and 34 participants for the *Reference room* were included in the analysis (30 and 33 respectively for the wearable light sensor). All participants gave written informed consent and study procedures were approved by the local ethical review board *Commission Cantonale d’éthique de la recherche sur l’être humain* (CER-VD, Lausanne, Switzerland).

The study was conducted at the École Polytechnique Féderale de Lausanne (EPFL; Switzerland) according to the tenets of the Declaration of Helsinki.

### Study protocol

Seven days prior to the office part of the study, participants were asked to maintain regular habitual bed- and wake times at home (within a self-selected range of +/−30 min) and do so for the entire study duration (i.e., four weeks; see overview on the study design on supplementary Figure S3). Compliance was verified by sleep logs and continuous actigraphy monitoring (ActTrust, Condor Instruments, São Paulo, Brasil). Participants were asked to consume alcohol, caffeine with moderation and not to take any medication (except for oral contraceptives). Habitual sleep onset and waketime (derived from activity watches) was on average at 00:14 ± 00:52 and at 8:01 ± 0:42 (hh:mm; mean ± SD). Participants’ sleep- and waketimes during the seven days before both office sessions did not significantly differ between the two conditions (*p* > 0.5; Table 2(a)). Midsleep time was derived from actigraphy recordings and calculated as the midpoint between habitual sleep times during the seven days prior to the respective laboratory condition (week 1 and week 3; Table 2(a)); it was on average at 04:07 ± 0:40 and there was no statistical difference between midsleep in week 1 and 3 (*p* = 0.23, Table 2(a)). These habitual midsleep times per room condition were used to align the data relative to elapsed time since midsleep. Sleep duration, calculated as the time between sleep onset and waketime during the 7 days before the study, was 07:41 ± 00:51 in the *Test room* and 07:53 ± 00:52 in the *Reference room* (*p* = 0.44).

During week 2 and 4, participants spent five consecutive days (8 h per day) in either the *Test room* or the *Reference room* in a randomized, counter-balanced order. We used a semi-randomization procedure, where each participant was assigned to a condition based on availability for the study and occupancy of the two rooms. None of the participants had an impact on the order of the condition or was aware of it. Retrosprectively, there was no statistical order effect on any outcome variable between the two room conditions (*p* > 0.4). Participants arrived approximately one hour after habitual waketime in either of the two office rooms. Individual protocols started on average at 8:43 ± 0:20 (i.e., 4:36 h after habitual midsleep) and were based on the averaged habitual sleep-and wake times of the seven days before (derived from activity watches). Sleep- and wake times were also monitored during the weeks in the office to verify compliance with the protocol. During the time in the office room, participants were allowed to quietly work, read, or listen to music. They could use their laptops and/or mobile phones. Participants also completed cognitive tasks and assessed their mood, wellbeing, and visual comfort at regular time intervals. Data on these endpoints will be reported elsewhere. Here, we focus on the circadian variables (i.e., melatonin, cortisol and DPG).

### Study measures

#### Vertical illuminance and spectral power distribution measured by stationary light devices in the office (Test room/Reference room)

Vertical illuminance (E_v_; lux) at the approximate eye level in the office was assessed by a stationary High Dynamic Range (HDR) vision sensor^78^, placed on a support next to the desk at a height of 1.20 m. Two identical devices were placed in the same position in both office rooms.

There is increasing evidence the non-visual effects of light on the circadian timing system, sleep and alertness cannot be solely explained by effects from photopic measures as a proxy for weighted cone sensitivity. Therefore, a new standard was recently developed based on the spectral sensitivity function of the third class of photoreceptors, the intrinsically photosensitive retinal ganglion cells (ipRGCs), which mediate non-visual light effects^12,79^. The metric [melanopic equivalent daylight (D65) illuminance] which was also used here in addition to photopic illuminance, is the weighted sensitivity function of ipRGCs for a given summated spectral power distribution of a light source relative to a daylight standard light source (D65)^80^.

To assess spectral power distribution (SPD) of the lighting, a spectrometer (Jeti Specbos 1201, JETI, Jena, Germany) was installed next to the vision sensor, on the same support. Since only one spectrometer device was available, SPD could only be monitored in one room at a time, therefore, all spectrally and irradiance derived measures were computed and analysed for 18 participants (both room conditions). Both E_v_ and SPD were continuously recorded at 5-min intervals and collapsed into hourly bins (aligned to habitual midsleep time for each participant) prior to analysis. From SPD measures, melanopic Equivalent (D65) Daylight Illuminance (EDI; lux) was calculated according to the CIE international standard^80,81^.

#### Individual light exposure measured by wearable light sensors at the eye level

In order to measure the light exposure across the entire waking periods at the eye level during weeks 2 and 4, the participants wore a light sensor mounted on glasses (‘Luxblick’^82,83^). They were asked to continuously wear the light sensor during the entire five days per condition, from the time when they woke up to when they went to bed, except for showering or some physical activity. The device recorded illuminance (photopic lux) at a 1 Hz resolution. All data were visually inspected (and excluded if necessary, see supplement), then collapsed into 24 hourly bins aligned to habitual midsleep time per condition. Accumulated individual illuminance (E_v_) across the waking day (per condition) was computed for each participant. The time when the accumulation curve reached its half-maximum (EC50) was determined on the individual light exposure profiles by using a non-linear logistic regression function following parametrization^84^.

#### Salivary samples for melatonin and cortisol assays

Saliva samples for melatonin and cortisol concentrations were regularly collected throughout the study (Figure S3). During the weeks in the office rooms (weeks 2 and 4), participants were asked to produce saliva samples on two days (day 2 and 4), at 11 occasions per day, i.e., every 60–120 min (from habitual wake time to bedtime, Figure S3). The first 1 or 2, and the last 5 samples were taken at home, under habitual evening light conditions and stored in the fridge until the next day. At arrival in the lab, all samples were immediately frozen at -20 °C and sent for radio-immuno assay (for details on the assay see supplement). The decision of collecting samples on day 2 and 4 of the weeks in the laboratory was made for practical reasons (e.g., to avoid additional visits to the laboratory for participants to return samples) and to keep the sampling periods at minimum. Melatonin secretion onset and offset times were calculated by linear interpolation by applying a threshold concentration of 3 pg/ml^85^ by using the software of Danilenko et al.^86^.

#### Skin temperature

Five small wireless temperature sensors (i-Buttons®, Maxim Integrated, USA) were taped on the back of both hands, feet and one below the left clavicle, to monitor skin temperature. Participants continuously wore the sensors during the laboratory part of the study (i.e., weeks 2 and 4). Skin temperatures were recorded at 5-min intervals. After downloading, the data was visually inspected, and artifacts excluded (see supplement). All data was collapsed into hourly bins prior to analysis (if not otherwise stated).

The distal–proximal skin temperature gradient (DPG) was calculated as the difference between the average temperature of hands and feet (distal) and the temperature at the clavicle (proximal)^87,88^. In order determine the time of the DPG rise in the evening for averaged 24-h DPGs profiles per participant, we added one standard deviation (SD) to the lowest DPG value in the time interval from 4 h before until 1 h after habitual sleep onset (using data averaged into 30-min bins). The exact time of the DPG rise was calculated by linear interpolation for each participant in each condition. For assessment of the DPG decline in the morning, one SD was subtracted from the highest DPG value in the time interval between 1 h before habitual waketime until 4 h after waketime (again using 30-min bins). The exact DPG decline time was then assessed by linear interpolation.

### Statistical analysis

The software SAS University Edition (SAS Institute Inc., Cary, NC, USA; v.9.4) was used to perform statistical analyses. We used a generalized linear model (GLIMMIX) to fit the data, with *lognormal* distribution when the data was not normally distributed (some of the data were linearly transformed from right to left skewed data prior to analysis according to). The fixed factors *condition* (*Test room* vs. *Reference room*) and *time* (for single time point comparisons) as well as their interactions were used. For repeated measurements, the factor *time* was used as repeated factor (with 11 or 18 levels respectively, or as stated in the text). For salivary melatonin and cortisol, data was averaged per condition for each participant and across days 2 and 4, because there was no statistically significant difference for melatonin onset on both days or an interaction with the factors *day* and *condition* (*p* > 0.5). Melatonin offset occurred earlier on day 4 than day 2 (*p* < 0.05), but there was no statistically significant difference between both conditions or an interaction between *day* and *condition* (see supplemental Table S3). We also plotted the individual differences between both conditions for all variables (except from cortisol) in the supplement (see Figure S7). Finally, *season* (3 levels) was added as covariate for all comparisons (see supplement). All data was aligned to elapsed time since habitual midsleep for each participant (derived from rest-activity cycles of the respective preceding week). Degrees of freedom were determined with the Satterthwaite approximation and post-hoc analysis performed by using the Tukey–Kramer test with *p* values adjusted for multiple comparisons or by using least square mean comparisons (with *p* values adjustments by using the false-discovery rate).

## Supplementary Information

Supplementary Information.
