# Supplementary material for: Optimized office lighting advances melatonin phase and peripheral heat loss prior bedtime
Source: Sci Rep. 2022 Mar 11;12:4267. doi: 10.1038/s41598-022-07522-8 (PMC8917232; doi:10.1038/s41598-022-07522-8)
Supplement: Supplementary file 1 — Supplementary Information. [file 41598_2022_7522_MOESM1_ESM.docx]

**SUPPLEMENTARY MATERIAL**

**Experimental set-up**

The experimental set-up consisted of two adjacent rooms on the second floor of the LESO experimental building (Figure S1a) on the campus of the Swiss Federal Institute of Technology Lausanne, Switzerland (EPFL; located at 46°31’N, Switzerland**).** Each room has a floor area of 17 m^2^ and large windows on the south façade including high performance anidolic daylighting systems^1^. The façade of the experimental building (LESO-PB, EPFL, Switzerland) is south-oriented (see supplementary Figure S1). The windows in the rooms are equipped with motorized venetian blinds (“Lamisol” and “Grinotex” metal blinds, Griesser AG, Aadorf, Switzerland).

We aimed to have the same electric lighting in both rooms. The ceiling light alone, however, was not sufficient to reach the target vertical illuminance E_v_ of 500 lux (see below), this is why we added also a floor-standing luminaire to both rooms. Specifically, the electric lighting fixture in each room consisted of two ceiling-mounted luminaires (Regent Lighting, Basel, Switzerland), and a floor-standing luminaire providing both direct and indirect light fluxes (model: Tweak Essential CLD LED, Regent Lighting, Basel, Switzerland), with dimmable LED lamps and a correlated colour temperature (CCT) of 4000 K. Spectral power distributions and α-opic quantities of the electric lighting are shown in Figure S2 and Table S2. The total connected electrical power in each room is 114 W (68 W for the free-standing luminaire and 23 W for each of the two ceiling-mounted luminaires).

The upper window area in the *Reference room* was covered in order to make the room similar to a standard office in terms of the window-to-wall ratio (WWR = ratio between the total glazed area and the total wall area). The WWR is equal to 39.5% in the *Test room* and 21% in the *Reference room*.

**Participants**

Study inclusion criteria were evaluated by the entrance questionnaire, the Munich Chronotype Questionnaire (MCTQ)^2^, the Horne-Ostberg questionnaire (HO) for assessment of morning-evening types^3^, the Epworth Sleepiness Scale (ESS)^4^ for scoring daytime sleepiness and the Pittsburgh Sleep Quality Index (PSQI)^5^ to assess habitual sleep quality. Inclusion criteria were: intermediate chronotype (MCTQ > 2.9 and < 5; HO > 30 and < 70); no excessive daytime sleepiness (ESS score < 10) and normal sleep quality (PSQI ≤ 5). Further inclusion criteria were: age between 19 and 30 years, in good health, i.e., non-smoker and absence of any psychiatric or medical disease and acute medication or drug intake (except for oral contraceptives). Exclusionary for study participation was shift work and recent travel across time-zones within the last three months before the study.

**Automated control system for blinds and electric lighting**

The automated controller for blinds and electric lighting was designed and programmed by the first author (M. Benedetti) and controlled via remotely accessible software (MATLAB ver. R2016b). It was programmed to run on a fixed algorithm throughout the study. The automated controller was designed to provide indoor lighting conditions that follow the dynamic daylight pattern, with minimum required illuminance levels varying dynamically throughout the day according to pre-set values (with a minimum E_v_ of 500 photopic lux in the morning, progressively increasing to at least 1000 lux at noon and decreasing again in the afternoon; maximum E_v_ should not exceed 2500 photopic lux). The aim of the automated controller is to ensure a vertical illuminance E_v_ (measured at the eye level) in the target range (see above), while also preserving visual comfort. To control for visual comfort the glare index Daylight Glare Probability (DGP)^6^ was kept in a comfortable range, i.e. lower than 35%. The DGP was computed by the embedded software of the High Dynamic Range vision sensor (see also ^7^). Such (comfortable) lighting conditions were enabled by closing the blinds when either absolute DGP or illuminance exceeded the upper threshold, and switching on electric lighting when vertical illuminance dropped below the minimum pre-set level due to insufficient illuminance from daylight. More details on the controller as well as the DGP results will be published elsewhere.

**Light exposure from wearable light sensors**

The wearable light sensor devices (‘Luxblick’^8,9^, TU Ilmenau, Germany) have an upper detection limit at ≈ 45’000 photopic lux (Dr. L. Wolf, personal communication). The wearable devices were tested for accurate measures prior the study. Data above such threshold and data collected when the device was removed (e.g., for shower) and during the night, were excluded prior to the analysis. Due to technical problems with the wearable light sensor, data from one participant in the *Test room* and one in the *Reference* room condition could not be used, which resulted in n = 30 and n = 33 respectively, for final analysis of individual E_v_. In addition, the DPG could not be calculated for one participant in the *Test* *room* because of sensor failure, hence DPG data from 30 participants in the *Test room* were included in the analysis.

**Quality controls for hormone assays**

For cortisol assays (ELISA ALPCO; Company, City/Country), the inter-assay coefficient of variance (CV) for high and low cortisol concentrations was 8.8% and 11.27% respectively; and for high cortisol concentrations, the intra-assay CV was 10.03%. Detection threshold was 4 ng/ml. For melatonin assays (Direct Saliva Melatonin RIA, order code: RK-DSM2, NovoLytiX GmbH, Witterswil, Switzerland), the inter-assay coefficient was 16.7% for low melatonin concentrations and 7.5% for high concentrations, while the intra-assay CV was 20.1% for low melatonin concentrations and 3.8% for high melatonin concentrations. Limit of quantification was 0.9 pg/ml and limit of detection 0.2 pg/ml. For one participant, the offset time in the *Test room* could not be assessed as it occurred before waketime.

**Skin and room temperature**

After the study, data was downloaded and visually inspected. Raw data larger than 42°C and lower than 22°C were excluded from analysis, as well as data when the sensors were removed (e.g. for shower or occasional sport activities). Room temperatures were continuously monitored and were on average slightly, but significantly higher in the *Reference room* than in the *Test room* (23.9 ± 1.6 °C vs. 23.1 ± 1.6 °C; p < 0.05).

**Seasonal differences and the impact of weather conditions and order of conditions**

For the analysis, seasons (i.e., winter, summer, spring and autumn) were determined as follows: the 1.5 months before and after winter and summer solstices (i.e., December 21 and June 21 respectively) were included in ‘winter’ and ‘summer’ respectively; the 1.5 months before and after both equinoxes (i.e., in spring and fall) were collapsed into ‘mid-seasons’.

There were no seasonal differences for E_v_ and melanopic EDI measured in the office room, nor a statistically significant seasonal difference between both room conditions for these variables (p > 0.1).

For light exposure assessed by wearable light sensors, there was significantly lower E_v_ in winter (see Figure S4; 395 ± 622 photopic lux) than both, summer (842 ± 1406 photopic lux) and mid-seasons (720 ± 1384 photopic lux; main effect of *season*, F_2,95_ = 6.28, p = 0.003), without significant differences between both conditions or interactions of *time* with *season* or *condition* (p>0.1). For analysis of the E_v_ from the wearable device only in the second half of the day (i.e., after 10 h from midsleep until bedtime), there was a significantly higher individual E_v_ in summer (689 ± 1152 photopic lux) than mid-seasons (560 ± 1317 photopic lux) and winter (204 ± 389 photopic lux; main effect of *season:* F_2,78_ = 7.6, p = 0.001). We also found a significantly earlier EC50 in winter [i.e., 7:54 ± 0:44 (hh:mm) since midsleep; main effect of *season*, F_2,46_ = 15.6, p < 0.0001] than in mid-seasons (9:16 ± 1:03 from midsleep) and summer (10:20 ± 1:33 from midsleep). To summarize, individual E_v_ was on average lowest and EC50 was earliest in winter, compared to mid-seasons and summer, without further differences for other variables or between room conditions.

There were no statistically significant seasonal differences for melatonin secretion profiles or skin temperatures, melatonin onset and offset times, and DPG decline times in the morning or phase angles between DPG decline and waketime (p > 0.1). For DPG rise times in the evening, we found a main effect of *season* (F_2,47_ = 4.8, p < 0.05) with earlier rise times in mid-seasons (18:27 ± 1:06 from midsleep) than winter (19:00 ± 0:48 from midsleep) and summer (19:22 ± 00:55 h from midsleep). Similarly, significant seasonal differences (p < 0.01) were also observed in the phase angles between DPG rise and sleep onset, which were longer in mid-seasons (1:39 ± 0:57) than winter (1:10 ± 0:49) and summer (0:39 ± 1:05).

In addition, we also tested for potential effects of weather: The amount of daily sunshine hours was used to classify the sky for each day of the study into three categories (clear, intermediate, overcast sky). The meteorological data was available from the local weather station. The analysis did not show any statistically significant difference in sunshine duration (used as a proxy of weather conditions) between both conditions (p>0.5). There were on average 5.03 ± 3.57 (mean ± SD) hours of sunshine per day in the *Test room* and 6.27 ± 3.66 hours in the *Reference room* ^11^.

Also, the order of conditions (i.e., *Reference-Test* or *Test-Reference*) did not have a statistically significant effect on any of the variables (p > 0.4, see also main text).

**SUPPLEMENTARY TABLES**

**Table S1: Participants' scores in entry questionnaires**

|  | **AGE** | **MCTQ** | **PSQI** | **HO** | **ESS** |
| --- | --- | --- | --- | --- | --- |
| **Mean** | 23.4 | 4.51 | 3.62 | 53.85 | 5.74 |
| **SD** | 3.2 | 0.72 | 1.30 | 6.79 | 2.64 |

**Table S1**. N = 34; 18 females; SD = standard deviation ; MCTQ = Munich Chronotype

Questionnaire; PSQI = Pittsburgh Sleep Quality Index; HO = Horne-Ostberg questionnaire; ESS = Epworth Sleepiness Scale.

**Table S2: Light quantities from electric lighting and α-opic quantities calculated from the spectral power distributions (SPD)**

| Luminaire | Irradiance [W/m^2^] | Photopic Illuminance [lux] | α-opic Irradiance [W/m^2^] | | | | | α-opic Equivalent Daylight (D65) Illuminance [lux] | | | | |
| --- | --- | --- | --- | --- | --- | --- | --- | --- | --- | --- | --- | --- |
|  |  |  | S-cone-opic | M-cone-opic | L-cone-opic | Rhodopic | Melanopic | S-cone-opic | M-cone-opic | L-cone-opic | Rhodopic | Melanopic |
| Ceiling-mounted | 0.55 | 180.90 | 0.09 | 0.23 | 0.29 | 0.18 | 0.15 | 106.30 | 159.11 | 180.52 | 124.10 | 111.58 |
| Floor-standing | 1.67 | 546.77 | 0.25 | 0.70 | 0.89 | 0.54 | 0.44 | 307.76 | 479.11 | 545.76 | 370.77 | 331.66 |

**Table S2.** The spectral power distributions (SPD) derived from the stationary spectrophotometer was similar in both rooms. Measurements were taken when either only the ceiling-mounted or the standing luminaire was on (without daylight), on a vertical plane at the approximate eye level of a person sitting at the desk, at 1.2 m height (see Figure S1 for room overview and position of sensors) by using the CIE toolbox^10^.

**Table S3: Timing of melatonin onset and offset on days 2 and 4**

|  | **Reference room** | | **Test room** | |  |
| --- | --- | --- | --- | --- | --- |
|  | **Day 2** | **Day 4** | **Day 2** | **Day 4** |  |
| **Melatonin onset** | 17.10 (1.58) | 16.90 (1.70) | 16.59 (1.80) | 16.74 (1.53) | ns |
| **Melatonin offset** | 5.19 (0.98) | 4.94 (1.08) | 5.18 (0.82) | 4.78 (1.05) | p = 0.03 |

**Table S3**. Timing of melatonin onset and offset on days 2 and 4 (for both room conditions; elapsed time since habitual midsleep in hours); means and SD (in brackets). P-values indicate significant differences for the main effect of day (i.e., day 2, 4). There was no statistically significant difference between the interaction with the factors ‘day’ and ‘condition’.

**Table S4: Sleep timing during the weeks in the laboratory**

|  | **During *Test room* Week (4 nights)** | **During *Reference room* Week (4 nights)** | **Differences between *Reference* and *Test room*** |
| --- | --- | --- | --- |
| Midsleep Time (h) | 3.79 (0.62) | 3.75 (0.66) | ns |
|  | 3:47 (00:37) | 3:44 (00:39) |  |
| Wake Time (h) | 11.26 (1.24) | 11.18 (1.19) | ns |
|  | 07:28 (01:14) | 07:25 (01:11) |  |
| Sleep Onset (h) | 27.84 (1.34) | 27.76 (1.47) | ns |
|  | 00:03 (01:20) | 00:00 (01:28) |  |
| Sleep Duration (h) | 7.42 (0.79) | 7.41 (0.71) | ns |
|  | 07:25 (00:47) | 07:24 (00:42) |  |

**Table S4**. Sleep timing for the week in the *Reference room* (column 2) and *Test room* (column 3) respectively (4 nights; for both conditions; elapsed time since real achieved midsleep in hours); mean times (decimal) and SD (in brackets) and corresponding clock times (hh:mm) in the respective lines below. There were no statistically significant differences between both weeks in the laboratory (ns = p > 0.1; see also Table 2a in the main text for more results).

**SUPPLEMENTARY FIGURES AND LEGENDS**

**Figure S1: Overview of the experimental study location**


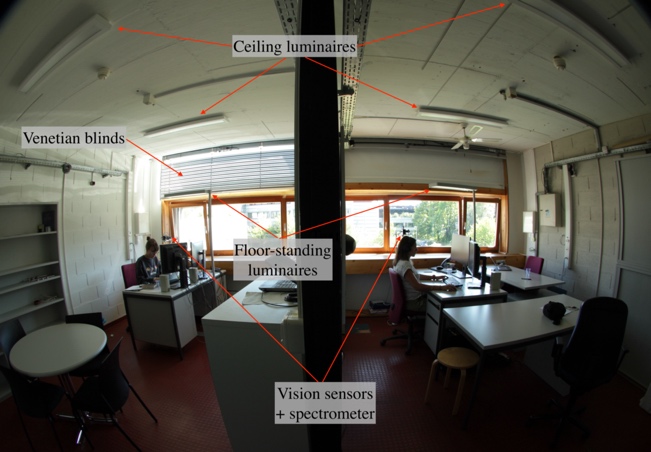

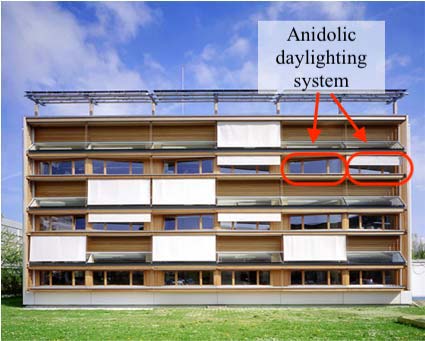
**S1a) S1b)**

**Figure S1.** a) The LESO-PB experimental building (south façade). The location of the two office rooms is marked by the red rectangles; red arrows indicate the upper part of the window (anidolic daylighting system) which always closed for the Reference room (right); b) Inside view of the two office rooms without electric lights on: Test room (left) and Reference room (right). Copyright pictures S1a and S1b: LESO-PB/EPFL (permission granted to Springer Nature Limited).

**Figure S2. Spectral power distributions from electric lighting in both rooms**

**S2a) S2b)**


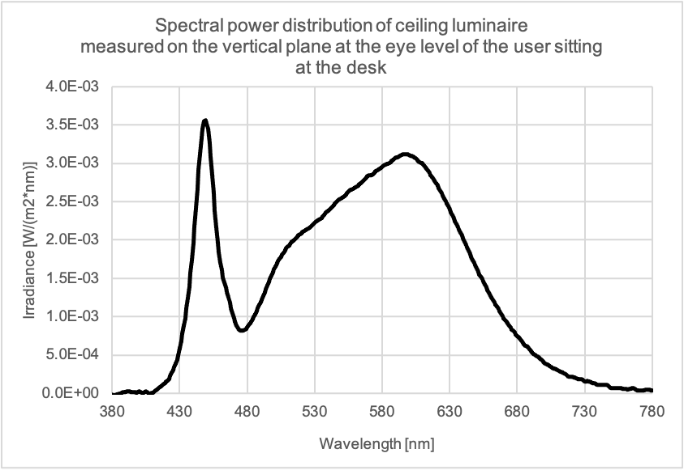

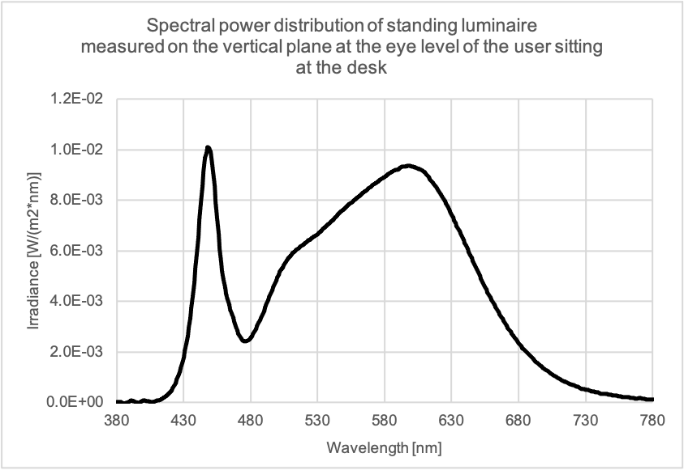


**Figure S2.** Spectral power distributions (SPD) of the two electric light sources, which were similar in both rooms: a) ceiling-mounted luminaire; b) floor-standing luminaire. The measurements were performed for each luminaire separately, in the absence of other light sources such as daylight. Measurements were taken on a vertical plane at the approximate eye level of a user sitting at the desk in the office (at 1.20 m height from the floor).

**Figure S3: Overview of the study protocol**


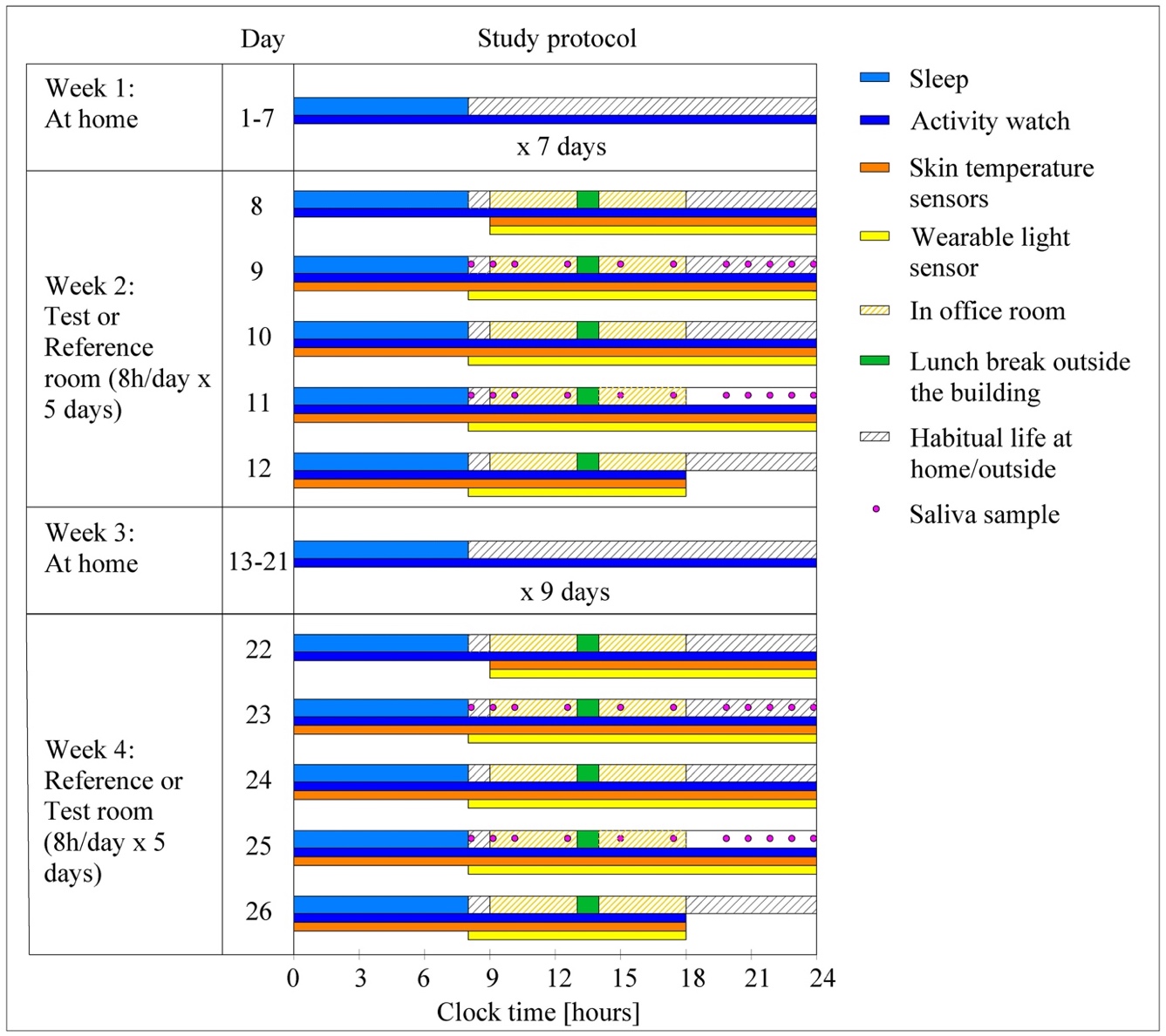


**Figure S3.** Overview of the 4-week study protocol: example for a participant with habitual waketime at 8:00. Sleep episodes are indicated by light blue rectangles; rectangles with grey stripes show the time when the participant could carry on his/her habitual life at home, at work or spending time outside. Rectangles with yellow stripes show the time spent in the office rooms (i.e., weeks 2 and 4) with 1-hour lunch break (outside the office; green rectangle). The times for the continuously worn activity watch are depicted by the dark blue rectangles and times when skin temperature sensors (i-buttons) were worn are shown by orange rectangles. The use of wearable light sensors is indicated with solid yellow rectangles (i.e., during weeks 2 and 4). Times for saliva samples (on day 2 and 4 of weeks 2 and 4) are shown by pink circles.

**Figure S4: Time course of illuminance (E_v_; from wearable devices) per season**

**
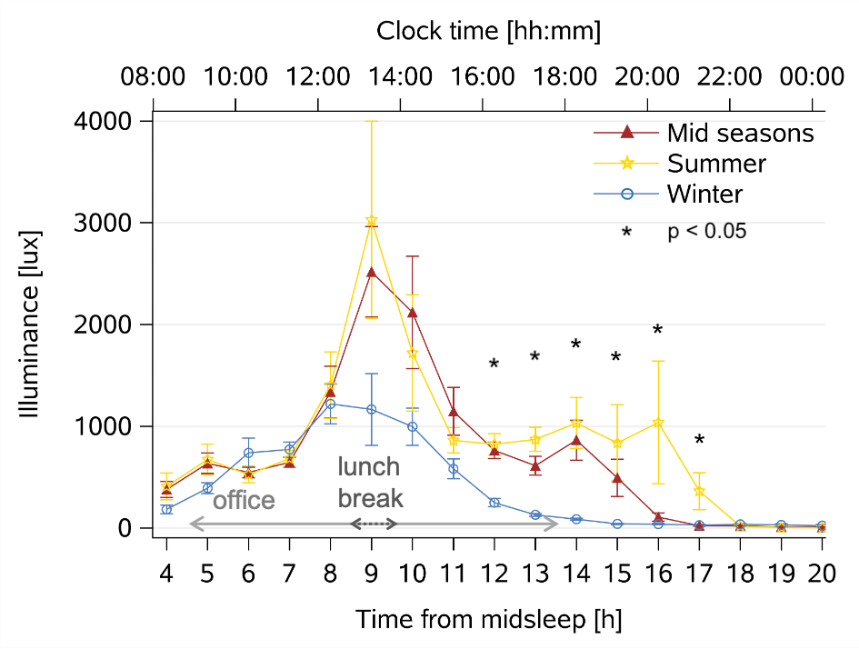
**

**Figure S4.** Time course of averaged individual illuminance (E_v_) from wearable light sensor devices across the entire waking period, averaged across 5 days per season [mean values ± SEM, winter (n = 8): blue circles and lines; mid-season (n = 17): red triangles and lines; summer (n = 9): yellow stars and lines; for statistics see supplementary text]. The x-axis at the bottom shows elapsed time since habitual midsleep, and the upper x-axis shows corresponding clock time (hh:mm). The horizontal arrows show the time in the offices and the lunch break (spent outside the office); * = significant differences between seasons (with p-values adjusted for multiple comparisons).

**Figure S5: Time courses of melatonin and cortisol concentrations**

**S5a) S5b)**


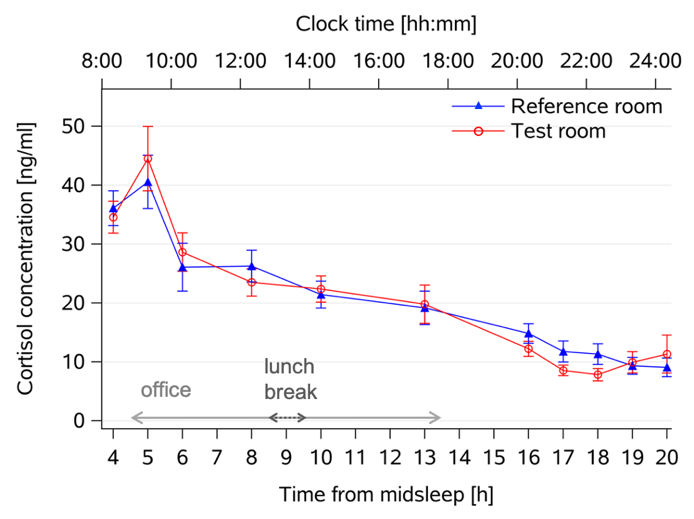

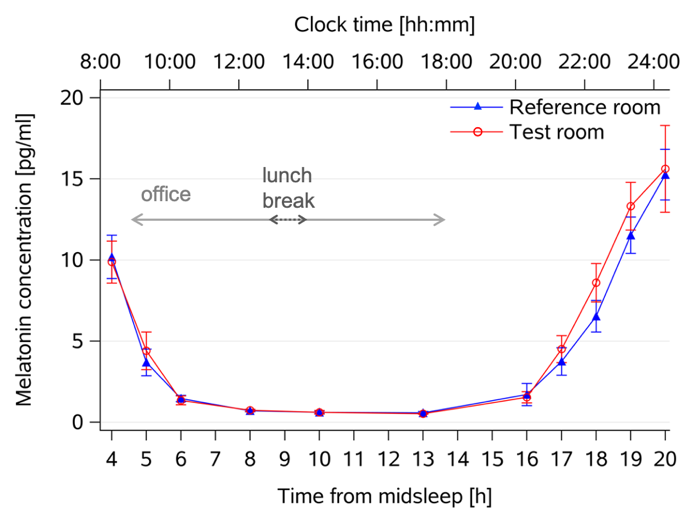


**Figure S5**. Time course of a) melatonin and b) cortisol concentrations (mean ± SEM) averaged across two days per room condition (*Test room*: red symbols and line, n = 31; *Reference room*: blue symbols and line, n = 34). Data is aligned to elapsed time since habitual midsleep (lower x-axis); corresponding clock time is shown on the upper x-axis. The horizontal arrows show the time in the offices (light grey arrows) and the lunch break (spent outside the office; dark grey arrows).

**Figure S6: Time course of distal-proximal skin temperature gradient**


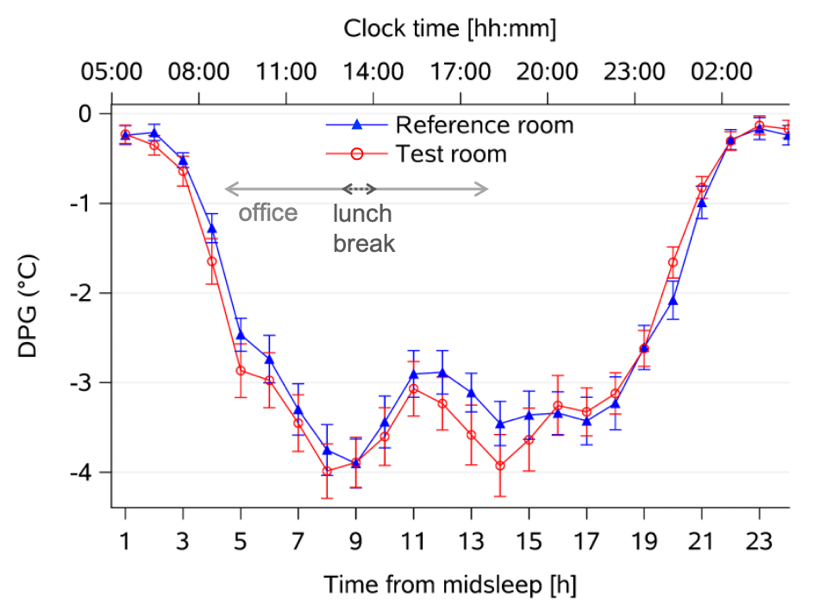


**Figure S6**: The mean 24-h DPG (°C) time course per condition [mean ± SEM; *Test room*: red circles and lines (n = 30); *Reference room*: blue triangles and lines (n = 34)]. The x-axis at the bottom depicts elapsed time since habitual midsleep (h) and the upper x-axis shows corresponding clock time (hh:mm). The horizontal arrows show the time in the offices and the lunch break (spent outside the office).

**Figure S7: Intra-individual differences**

**
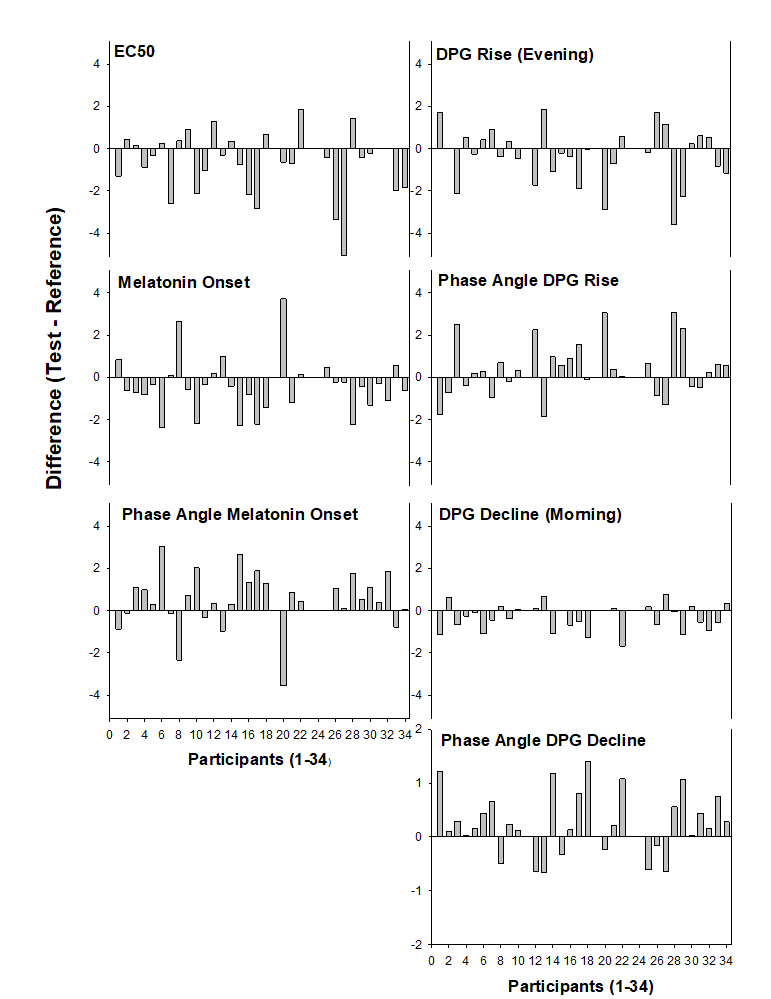
**

**Figure S7**. Intra-individual differences (grey bars) between the *Test room* and the *Reference room* (*Test room* minus *Reference* *room*) for the EC50, melatonin onset, phase angle between melatonin and sleep onset, DPG rise in the evening, phase angle of DPG and sleep onset, DPG decline in the morning and the phase angle between DPG decline and waketime (all differences derive from elapsed hours since habitual midsleep time; n = 34). Positive numbers indicate greater values or later times for the *Test* than the *Reference* condition.

**Figure S8: Intra-individual differences of evening light exposure (last 5 h before bedtime)**

**
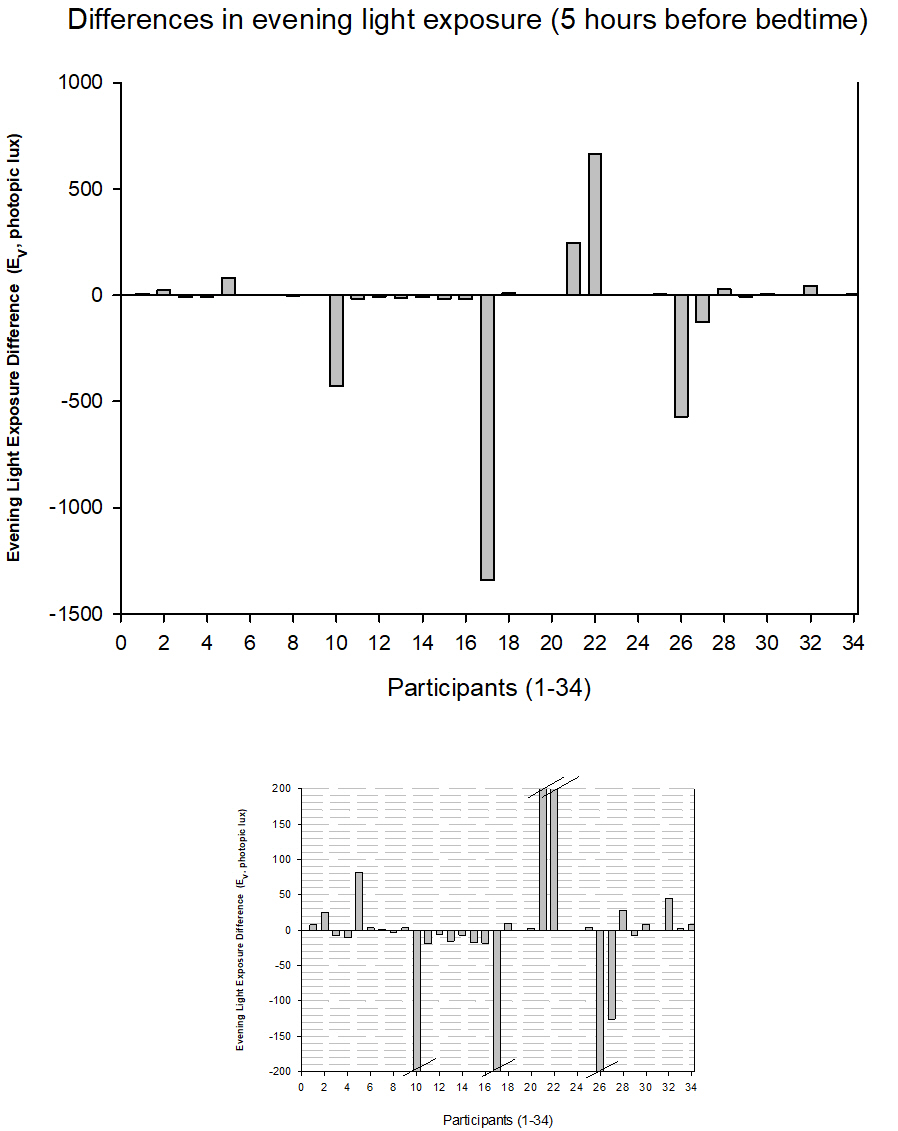
**

**Figure S8.** Intra-individual differences for evening light exposures during the 5 hours before bedtime derived from wearable devices (average of 4 days per participant, *Test room* minus *Reference room*). The upper graph shows a larger y-scale, whereas in the lower graph the largest differences are cut (indicated by the lines above or below the bars). The majority of participants had small differences between both conditions (n = 29). A positive number indicate higher E_v_ in the *Test room*.

**References**

1. Scartezzini, J.-L. & Courret, G. Anidolic daylighting systems. *Sol. Energy* **73**, 123–135 (2002).

2. Roenneberg, T., Wirz-Justice, A. & Merrow, M. Life between clocks: daily temporal patterns of human chronotypes. *J. Biol. Rhythms* **18**, 80–90 (2003).

3. Horne, J. A. & Ostberg, O. A self assessment questionnaire to determine Morningness Eveningness in human circadian rhythms. *Int. J. Chronobiol.* **4**, 97–110 (1976).

4. Johns, M. W. A new method for measuring daytime sleepiness: The Epworth sleepiness scale. *Sleep* **14**, 540–545 (1991).

5. Buysse, D. J., Reynolds, C. F., Monk, T. H., Berman, S. R. & Kupfer, D. J. The Pittsburgh Sleep Quality Index: a new instrument for psychiatric practice and research. *Psychiatry Res.* **28**, 193–213 (1989).

6. Wienold, J. & Christoffersen, J. Evaluation methods and development of a new glare prediction model for daylight environments with the use of CCD cameras. *Energy Build.* **38**, 743–757 (2006).

7. Motamed, A., Deschamps, L. & Scartezzini, J.-L. On-site monitoring and subjective comfort assessment of a sun shadings and electric lighting controller based on novel High Dynamic Range vision sensors. *Energy Build.* **149**, 58–72 (2017).

8. Hubalek, S., Zöschg, D. & Schierz, C. Ambulant recording of light for vision and non-visual biological effects. *Light. Res. Technol.* **38**, 314–321 (2006).

9. Wolf, S. LuxBlick — Mobile Langzeitaufzeichnung von Beleuchtungsstärke und circadianer Bestrahlungsstärke am Auge. in *Lux junior - Internationales Forum für den Lichttechnischen Nachwuchs* (2009).

10. Commission Internationale de l’Eclairage. CIE System for Metrology of Optical Radiation for ipRGC-Influenced Responses to Light (CIE S 026/E:2018). (2018).

11. Benedetti, M. Integrating Non-Visual Effects Of Light In The Automated Daylight

Responsive Control Of Blinds And Electric Lighting. EPFL PhD Thesis no. 8414, Ecole Polytechnique Fédérale de Lausanne, 1–192 (November 2021).
